# Precision Therapeutics in Lennox–Gastaut Syndrome: Targeting Molecular Pathophysiology in a Developmental and Epileptic Encephalopathy

**DOI:** 10.3390/children12040481

**Published:** 2025-04-08

**Authors:** Debopam Samanta

**Affiliations:** Division of Child Neurology, University of Arkansas for Medical Sciences, Little Rock, AR 72202, USA; dsamanta@uams.edu; Tel.: +1-(434)-806-1441; Fax: +1-(501)-364-6077

**Keywords:** Lennox–Gastaut syndrome, precision medicine, epileptic encephalopathy, gene therapy, channelopathies, antisense oligonucleotides, genetic epilepsy, molecular targeted therapy, neurodevelopmental disorders, pharmacogenomics

## Abstract

Lennox–Gastaut syndrome (LGS) is a severe childhood-onset developmental and epileptic encephalopathy characterized by multiple drug-resistant seizure types, cognitive impairment, and distinctive electroencephalographic patterns. Current treatments primarily focus on symptom management through antiseizure medications (ASMs), dietary therapy, epilepsy surgery, and neuromodulation, but often fail to address the underlying pathophysiology or improve cognitive outcomes. As genetic causes are identified in 30–40% of LGS cases, precision therapeutics targeting specific molecular mechanisms are emerging as promising disease-modifying approaches. This narrative review explores precision therapeutic strategies for LGS based on molecular pathophysiology, including channelopathies (*SCN2A*, *SCN8A*, *KCNQ2*, *KCNA2*, *KCNT1*, *CACNA1A*), receptor and ligand dysfunction (GABA/glutamate systems), cell signaling abnormalities (mTOR pathway), synaptopathies (*STXBP1*, *IQSEC2*, *DNM1*), epigenetic dysregulation (*CHD2*), and CDKL5 deficiency disorder. Treatment modalities discussed include traditional ASMs, dietary therapy, targeted pharmacotherapy, antisense oligonucleotides, gene therapy, and the repurposing of existing medications with mechanism-specific effects. Early intervention with precision therapeutics may not only improve seizure control but could also potentially prevent progression to LGS in susceptible populations. Future directions include developing computable phenotypes for accurate diagnosis, refining molecular subgrouping, enhancing drug development, advancing gene-based therapies, personalizing neuromodulation, implementing adaptive clinical trial designs, and ensuring equitable access to precision therapeutic approaches. While significant challenges remain, integrating biological insights with innovative clinical strategies offers new hope for transforming LGS treatment from symptomatic management to targeted disease modification.

## 1. Introduction

Lennox–Gastaut syndrome (LGS) is a childhood-onset developmental and epileptic encephalopathy (DEE) that represents 10% of all epilepsy cases with onset before the age of 5 [1]. The overall prevalence of LGS is approximately 26 per 100,000 people, and it is more commonly seen in males than in females [2,3]. It is characterized by (1) multiple drug-resistant seizure types, with tonic seizures as a hallmark feature; (2) cognitive and often behavioral impairments; and (3) distinctive electroencephalographic (EEG) patterns, including diffuse slow spike-and-wave activity and generalized paroxysmal fast activity [4,5]. LGS arises from diverse etiologies—including structural, genetic, infectious, metabolic, and immune causes—ultimately disrupting bilaterally distributed brain networks [4].

Current LGS treatment primarily targets seizure management through antiseizure medications (ASMs), dietary therapy, epilepsy surgery, and neuromodulation, with limited impact on disease progression [6,7,8,9]. Most therapies achieve only modest seizure reduction and offer even less benefit for cognitive and behavioral comorbidities, which significantly affect quality of life and, in some cases, cause substantial adverse effects [10].

Precision therapeutic approaches represent an emerging frontier, particularly as 30–40% of LGS cases have an identifiable genetic etiology [11,12,13,14,15,16]. Advances in understanding LGS-associated genes at the cellular and network levels enable classification of patients into biologically distinct subgroups with differing pathophysiology and treatment responses. Tailoring interventions based on these characteristics may improve efficacy while reducing adverse effects associated with nonspecific treatments [17]. Furthermore, many individuals with LGS initially present with early infantile epileptic encephalopathy or infantile epileptic spasms syndrome before evolving into LGS [4]. A key advantage of disease-modifying therapies is their potential to target pathogenic mechanisms early in the disease course, potentially preventing the progression of some infantile epileptic encephalopathies to LGS.

This narrative review explores precision therapeutic strategies based on specific monogenic causes and disease mechanisms relevant to LGS. A comprehensive literature search (PubMed, MEDLINE, ClinicalTrials.gov, conference abstracts from the American Academy of Neurology and American Epilepsy Society, and gray literature) was conducted through 19 February 2025 to identify established ASMs, repurposed and novel drugs, as well as various gene therapy approaches with potential relevance to LGS. Given that over 900 monogenic causes of DEEs have been identified—implicating diverse cellular components such as ion channels, receptors, synaptic proteins, signaling pathways, metabolic processes, and epigenetic regulators—this review discusses current and emerging precision therapeutics based on shared molecular mechanisms and the pathophysiology of select genes associated with LGS [17] (Table 1).

## 2. Channelopathies

Voltage-gated sodium (Na^+^), potassium (K^+^), and calcium (Ca^2+^) channels play a critical role in neuronal excitability and network dysfunction in LGS [18]. While much progress in precision therapy has been made in *SCN1A* mutations, primarily associated with Dravet syndrome, this section focuses on precision therapies targeting monogenic channelopathies more commonly linked to LGS, including *SCN2A* and *SCN8A* (sodium channels), *KCNQ2*, *KCNA2*, and *KCNT1* (potassium channels), and *CACNA1A* (calcium channels) [19,20].

### 2.1. SCN2A and SCN8A

The *SCN2A* and *SCN8A* genes encode the neuronal voltage-gated sodium channels NaV1.2 and NaV1.6, respectively [21,22]. NaV1.2 (*SCN2A*) is predominantly expressed in excitatory neurons during early development and is later functionally replaced in some aspects by NaV1.6 (*SCN8A*), which is widely distributed in both excitatory and inhibitory neurons and plays a crucial role in neuronal excitability and action potential propagation [23]. Pathogenic variants in these genes cause DEEs with distinct phenotypes depending on whether they result in gain-of-function (GOF) or loss-of-function (LOF) effects. *SCN2A*-related epilepsy typically manifests in early childhood and spans a broad spectrum from self-limited epilepsy to severe early infantile epileptic encephalopathy, which can evolve into infantile epileptic spasms syndrome and LGS, primarily in cases associated with GOF variants [24]. In contrast, seizure onset after three months to one year is often linked to LOF variants, which may also cause developmental delays and autism without epilepsy [25]. Similarly, *SCN8A*-related epilepsy exhibits a range of phenotypes, with LGS associated with GOF variants, though epilepsy onset is typically later in infancy compared to *SCN2A* [26].

GOF variants in *SCN2A* and *SCN8A* are specifically responsive to sodium channel blockers (SCBs) such as carbamazepine and phenytoin [25,27]. These SCBs primarily inhibit the fast inactivation of sodium channels, reducing transient sodium currents that drive action potential firing [28]. However, they do not significantly affect persistent sodium currents, which prolong neuronal depolarization and contribute to hyperexcitability and seizures [29]. Additionally, blocking transient currents can lead to dose-dependent side effects, including ataxia, sedation, and cognitive impairment [28].

Because persistent sodium current reduction by traditional SCBs may be insufficient at clinically relevant doses, alternative approaches have been explored. One study showed that Phrixotoxin-3, a sodium channel blocker relatively specific for Nav1.2, was particularly effective in reducing hyperexcitability in networks of human neurons expressing a GOF variant, whereas traditional sodium channel blockers like phenytoin were ineffective [30]. Cenobamate, an approved ASM, has been investigated for its ability to modulate persistent sodium currents. In a study of 12 patients with presumed GOF *SCN8A* variants, cenobamate treatment for an average of 17 months resulted in a meaningful reduction in countable motor seizures in 10 patients (83%) [31]. Among them, six achieved >70% seizure reduction, including two who achieved seizure freedom [31]. Relutrigine (PRAX-562) is a first-in-class small molecule that preferentially inhibits persistent sodium currents [32,33]. A Phase 2 randomized controlled trial (NCT05818553; EMBOLD) involving 16 patients with *SCN2A* and *SCN8A* variants demonstrated good tolerability, with a placebo-adjusted monthly motor seizure reduction of 46% during the double-blind phase [34]. Over 30% of patients achieved seizure freedom, with notable improvements in alertness, communication, and seizure severity. In the long-term extension, patients experienced a median 77% seizure reduction [34].

Another novel compound, NBI-921352, selectively inhibits NaV1.6 and has shown efficacy in preventing seizures in mouse models at lower brain and plasma concentrations than traditional SCBs [35]. As a state-dependent inhibitor, NBI-921352 preferentially targets inactivated channels, stabilizing inactivation and reducing NaV1.6-mediated resurgent and persistent currents while sparing resting channels [35]. This isoform-selective profile allows robust inhibition of excitatory pyramidal neurons while preserving inhibitory interneurons, which predominantly express NaV1.1 [35]. Clinical trials for *SCN8A*-related DEEs (NCT04873869) are currently underway. Beyond these established ASMs and emerging therapies, amitriptyline, carvedilol, and nilvadipine have also demonstrated concentration-dependent inhibition of sodium channel currents in cellular GOF *SCN8A* models [36].

Additionally, the ketogenic diet has shown efficacy in some *SCN2A*-related DEEs [37,38,39,40].

Antisense oligonucleotides (ASOs) have been explored for *SCN2A* and *SCN8A* GOF variants. In a mouse model, central administration of a gapmer ASO targeting *Scn2a* mRNA significantly reduced spontaneous seizures and extended lifespan [41]. A clinical trial (the EMBRAVE study; NCT05737784) is currently evaluating elsunersen (PRAX-222), an ASO designed to downregulate NaV1.2 expression via RNase H-mediated degradation of *SCN2A* mRNA [42]. In this study, participants (ages 2–18 years) received elsunersen intrathecally at ≥4-week intervals for up to 13 weeks [43]. Four evaluable participants, each receiving four doses, demonstrated a 43% median seizure reduction from baseline, along with a 48% relative increase in seizure-free days. No significant adverse effects were reported. In a study, conditional knock-in mice carrying a GOF Scn8a variant were treated with ASOs that reduced Scn8a/NaV1.6 expression by 25–50%, resulting in a delay in spontaneous seizure onset and lethality [44].

### 2.2. KCNQ2

The *KCNQ2* gene encodes the Kv7.2 potassium channel, which is crucial for generating the neuronal muscarine-regulated M-type current [17]. Loss-of-function (LOF) *KCNQ2* variants, particularly dominant-negative missense mutations that reduce potassium currents in Kv7.2, are typically responsible for *KCNQ2*-related early infantile developmental and epileptic encephalopathy. *KCNQ2*-DEE is characterized by frequent, primarily tonic seizures with focal motor and autonomic features, beginning within the first week of life. While seizures usually resolve between 9 months and 4 years of age, some cases progress to a LGS-like phenotype [45].

Sodium channel blockers (SCBs) have demonstrated efficacy in *KCNQ2*-DEE, likely due to the colocalization of potassium and sodium channels at the neuronal membrane, allowing responsiveness to SCBs [46]. One study reported that 30–50% of patients with *KCNQ2* variants achieved seizure freedom with early treatment using carbamazepine or phenytoin [47].

A more targeted approach involves potassium channel openers like ezogabine (retigabine). In one study, ezogabine improved seizure control and developmental outcomes in three out of four patients treated before six months of age and in two out of seven patients treated later, with no serious adverse effects [48]. Another study reported that all five patients with daily seizures experienced a >50% reduction in seizure frequency within 1–2 weeks of treatment [49]. Developmental gains in alertness, vocalization, and motor skills were observed in all eight treated patients, with urinary retention being the only reported side effect in two cases.

Despite its potential benefits, ezogabine was withdrawn from the market due to concerns about abnormal skin and eye pigmentation [50]. Several modified potassium channel openers are currently in development. Although the clinical trial of XEN496, an ezogabine-like drug for *KCNQ2*-related DEE (NCT04639310), was terminated, other potassium channel openers, such as XEN1101 and BHV-7000, are being evaluated in broader epilepsy populations [51,52].

### 2.3. KCNA2

Gain-of-function (GOF) and mixed GOF–LOF variants in *KCNA2*, which encode the Kv1.2 channel subunit, are implicated in DEEs, including LGS [17]. The potassium channel blocker 4-aminopyridine has been shown to counteract GOF-related defects by reducing current amplitudes, shifting steady-state activation, and enhancing neuronal firing. In an *n*-of-1 trial conducted across nine centers, 9 out of 11 patients with *KCNA2* variants benefited from 4-aminopyridine treatment [53]. Improvements were noted in seizure control (except for generalized tonic–clonic seizures) as well as in gait, ataxia, speech, and cognition, with good tolerability.

### 2.4. KCNT1

Heterozygous de novo pathogenic variants in *KCNT1*, which encode a sodium-activated potassium channel, are associated with a spectrum of DEEs. While epilepsy in infancy with migrating focal seizures and sleep-related hypermotor epilepsy are the most frequently observed phenotypes, LGS has also been reported [54]. Functional studies indicate that GOF mutations lead to increased potassium current amplitude, correlating with phenotypic severity. Inhibitory neurons appear particularly vulnerable, as increased potassium current results in decreased neuronal firing and a subsequent loss of inhibition [55].

Quinidine, a non-specific potassium channel blocker, has shown variable benefits in *KCNT1*-related epilepsy. One case report described a patient with LGS who experienced a 75% reduction in tonic seizures and a 55% decrease in epileptiform discharges after eight months of quinidine therapy, though other seizure types remained unaffected [54]. Another approach utilizes gene silencing with ASOs. In a study using a Kcnt1 gapmer ASO, a single intracerebroventricular bolus injection administered to symptomatic mice at postnatal day 40 significantly decreased seizure frequency, improved behavioral abnormalities, and prolonged overall survival [56].

### 2.5. CACNA1A

The *CACNA1A* gene encodes the pore-forming α1 subunit of the voltage-gated P/Q-type calcium channel (Cav2.1), which regulates intracellular calcium influx [17]. Mutations in *CACNA1A* are well-known causes of familial hemiplegic migraine, episodic ataxia type 2, and spinocerebellar ataxia type 6. However, both GOF and LOF de novo mutations in *CACNA1A* have also been associated with various epilepsy syndromes, including LGS [57].

For *CACNA1A*-related disorders, certain medications have shown efficacy, including lamotrigine, topiramate, levetiracetam, acetazolamide, and calcium channel blockers such as flunarizine and verapamil [58]. Additionally, a case report described a positive therapeutic response to carbamazepine [59].

## 3. Receptor- and Ligand-Mediated Dysfunction

Neurotransmission depends on the precise interaction between receptors and their ligands to maintain neural network stability. In LGS, mutations affecting GABAergic and glutamatergic receptors or disruptions in neurotransmitter synthesis, release, and transport can lead to excessive excitation or impaired inhibition, contributing to epilepsy and cognitive dysfunction.

### 3.1. GABA/GABA Receptor Dysfunction

As the principal inhibitory neurotransmitter in the brain, GABA plays a crucial role in seizure control. Dysfunction in various GABA receptor subunit genes (*GABRA1*, *GABRA2*, *GABRB2*, *GABRB3*) has been implicated in DEEs. Two patients with LGS/DEE and *GABRB3* or *GABRA1* LOF variants showed remarkable seizure and cognitive improvement with vinpocetine, a positive modulator of GABA (α1β3γ2) receptors [60,61]. However, in GOF variants, vinpocetine may exacerbate symptoms, as observed with vigabatrin in *GABRB3* GOF cases [62].

Extrasynaptic GABA-related DEEs, such as those linked to *SLC6A1*—which encodes GABA transporter 1 (hGAT1), responsible for clearing GABA from the synaptic cleft—have been associated with LGS [63]. Broad-spectrum ASMs, including valproic acid, lamotrigine, and benzodiazepines, have shown efficacy. In a prior study, 20 of 31 patients became seizure-free with ASMs, with valproic acid being the most effective, followed by lamotrigine and ethosuximide [64]. Additionally, the ketogenic diet has demonstrated effectiveness for myoclonic-atonic seizures [65].

### 3.2. Glutamate Receptor Dysfunction

Ionotropic glutamate receptors, including NMDA and AMPA receptors, are key mediators of fast excitatory neurotransmission [66]. Among *GRIN*-related disorders, *GRIN2B* variants are most commonly associated with LGS [67]. For GOF variants in *GRIN1*, *GRIN2A*, and *GRIN2D*, limited reports suggest potential benefit from memantine, an NMDA receptor (NMDAR) blocker [68].

Radiprodil, a selective negative allosteric modulator of NMDAR subtype 2B, was evaluated in 15 patients with GOF variants in a Phase 1 Honeycomb study (NCT05818943) [69]. This trial demonstrated a median 86% reduction in seizure frequency, with 71% of patients experiencing at least a 50% reduction in countable motor seizures and 43% achieving >90% seizure reduction. Notably, one patient became seizure-free following treatment. For null variants in *GRIN2B* and *GRIN2A*, an *n*-of-1 trial using L-serine, an essential amino acid and NMDAR co-agonist, showed benefits in seizure control in four of nine patients and behavioral improvements in eight of nine patients [70]. However, as these variants typically do not result in an LGS phenotype, further discussion is beyond the scope of this review.

## 4. Synaptopathies

Synaptopathies—disorders resulting from dysfunction in synaptic proteins—can contribute to LGS by disrupting the excitatory/inhibitory balance [17]. These disruptions arise from defects in synaptic vesicle cycling and release, synaptic scaffolding, receptor trafficking, and impaired synaptic plasticity and development [17]. Below, we discuss emerging therapeutics targeting some common genetic causes of these synaptopathies.

### 4.1. STXBP1

Syntaxin-binding protein 1 (STXBP) encephalopathy, often characterized by early-onset seizures, can evolve into LGS. The median age of seizure onset is typically around six weeks. STXBP1 is a membrane transport protein that interacts with SNARE proteins, playing a critical role in synaptic vesicle release at presynaptic active zones [46]. Pathogenic missense variants of *STXBP1* cause a dominant-negative effect, destabilizing the protein, promoting aggregation, and depleting functional wild-type Munc18-1 protein [71].

Chemical chaperones, such as 4-phenylbutyrate, sorbitol, and trehalose, have shown promise in reversing deficits caused by these mutations in both in vitro and in vivo models [72]. These compounds work by stabilizing misfolded proteins, reducing aggregation, preventing harmful interactions, and promoting proper protein folding and translocation. In one study, 10 weeks of 4-phenylbutyrate treatment led to a 60% seizure reduction in patients with STXBP1 variants. Side effects included metabolic acidosis, honey-like odor, sedation, and anorexia [73].

Cannabidiol (CBD) products were noted to be effective in patients with STXBP1 gene mutations [74]. Though the exact mechanism remains unclear, CBD acts as a functional antagonist of GPR55 (G protein-coupled receptor 55) and may regulate neurotransmitter release. Additionally, the ketogenic diet has shown efficacy in STXBP1-related seizures [75,76].

Levetiracetam (LEV), which targets the synaptic vesicle protein SV2A, has demonstrated some benefit in STXBP1 patients. In a study comparing 26 patients on LEV with 10 patients on other antiseizure medications, 88% of LEV-treated patients achieved a >50% seizure reduction at 6 months, compared to 50% of those on alternative treatments [75]. However, while LEV reduced seizure frequency, no significant difference was found in seizure freedom rates [75]. Larger-scale studies, like one by Xian et al., suggest that LEV may not be as effective as once believed for seizure control in STXBP1-related disorders, as it showed a low odds ratio for seizure reduction and freedom [75].

Medications targeting serotonin receptors have also been considered. Screening antiseizure drugs in a zebrafish model of STXBP1-related disorders identified clemizole and trazodone as reducing ictal events [77]. These findings suggest that drugs with serotonin receptor-binding properties, such as fenfluramine, could prove effective, though further research is needed (NCT05232630).

Additionally, research has shown that physiological microRNAs can target the STXBP1 transcript, significantly reducing its transcription. A study found that microRNAs encoded by the Herpes Simplex Virus 1 (HSV-1) Latency-Associated Transcript (LAT) could regulate STXBP1 expression [78]. MicroRNAs (miRNAs), which are small, non-coding RNA molecules (~22 nucleotides), regulate gene expression at the post-transcriptional level by binding to complementary sequences in the 3′ untranslated region (3′UTR) of mRNAs. Therapies targeting miRNAs may help increase the levels of STXBP1-encoded Munc18-1 protein, offering potential benefits for patients [79].

### 4.2. IQSEC2

IQSEC2 encodes a guanine nucleotide exchange factor that interacts with ARF6 and other GTPases, playing a crucial role in AMPA receptor trafficking and synaptic plasticity [80]. This X-linked disorder is associated with intellectual disability and epilepsy, including LGS [81]. In a mouse model, behavioral rescue was achieved with a positive AMPAR modulator [82]. Ongoing research is investigating various AMPA modulators, such as perampanel, methylphenidate, and aniracetam, in zebrafish models [83]. Additionally, RNA sequencing of hippocampi from a mouse model revealed dysregulation of genes related to thyrotropin-releasing hormone, suggesting a potential precision therapy approach targeting these pathways [84].

### 4.3. DNM1

Dynamin 1 (DNM1) encodes a GTPase involved in synaptic vesicle fission for receptor-mediated endocytosis at the presynaptic plasma membrane [85]. Pathogenic variants in *DNM1* lead to severe DEEs due to a dominant-negative effect [86]. The ketogenic diet has shown efficacy in a small subset of patients, while some rare cases have responded to valproic acid, clobazam, or vigabatrin [87].

In a mouse model with the DNM1 mutation, dysfunctional endocytosis, altered excitatory neurotransmission, and seizure-like phenotypes were observed. These phenotypes were corrected at the cellular, circuit, and in vivo levels by the drug BMS-204352, which accelerates endocytosis [88]. RNA interference (RNAi) has also emerged as a potential strategy to silence toxic gene expression [71]. In one study, a DNM1-targeted microRNA delivered via self-complementary Adeno-associated virus (AAV) 9 to homozygous mice resulted in reduced seizures and improved cellular features in brain histology [89].

Given the complexity of DNM1-related disorders, a dual approach may be required—eliminating harmful mutant gene products while restoring normal gene function. A study using an AAV9-based vector in newborn mice combined an RNAi sequence targeting the mutant DNM1 mRNA with a replacement DNM1 cDNA resistant to the RNAi [90]. This approach resulted in improved electrophysiological recordings and normalized gene expression in transcriptome analysis, highlighting the potential of combinatory therapies [90].

## 5. Cell Signaling Dysfunction

Several cell signaling pathways play critical roles in epilepsies, with the mechanistic target of rapamycin (mTOR) pathway being the most prominent. The mTOR signaling pathway regulates essential cellular processes such as metabolism, growth, proliferation, and survival. Germline variants in *TSC1* and *TSC2* cause tuberous sclerosis complex (TSC), a multisystem disorder characterized by cortical tubers and primarily focal epilepsy. However, infantile epileptic spasms syndrome is a common initial presentation, and some patients may evolve into the LGS phenotype. Additionally, somatic mutations in cell signaling genes—or a combination of somatic and germline mutations—can contribute to malformations of cortical development, another significant cause of LGS.

Precision therapeutics for cell signaling primarily focus on early and effective treatment of infantile seizures to possibly prevent progression to LGS [91]. Vigabatrin (VGB) is widely considered first-line monotherapy for TSC-associated IESS, with response rates ranging from 67 to 96% in open-label studies and randomized controlled trials [91]. However, it remains unclear why VGB is more effective for TSC-associated infantile spasms than for spasms due to other etiologies. Preemptive VGB treatment at the first sign of epileptiform activity on EEG has been shown to reduce the risk of developing infantile spasms [92]. While the risk of developing other seizures or drug-resistant epilepsy (possibly progressing to LGS) varies across studies, no significant changes in cognition or autism have been observed [92,93,94]. More relevant to this review is the uncertainty regarding whether TSC-related LGS is more responsive to VGB. While one study reported that 85% of children with LGS (not specific to TSC) experienced a 50–100% reduction in seizure frequency, other studies have suggested a possible worsening of seizures in LGS following VGB treatment [95,96]. Further investigation is needed to clarify this potential relationship.

For a more targeted approach, everolimus, an mTOR inhibitor, was supported by the phase 3 EXIST-3 study for use in TSC-associated seizures [97]. Another mTOR inhibitor, sirolimus, has been evaluated for TSC-associated seizures, with one study showing that early treatment with sirolimus, particularly before 6 months of age, delayed seizure onset and the development of infantile spasms [98]. However, the specific efficacy of everolimus and sirolimus in preventing the development of LGS or treating various seizure types associated with LGS remains unclear. Additionally, CBD has demonstrated efficacy in TSC-associated seizures in children over 1 year old, as well as for LGS, though the mechanistic basis of its efficacy remains unclear [99]. Some studies suggest that CBD may influence the mTOR pathway, potentially upregulating or downregulating mTOR, depending on the context [100]. Lastly, further gene therapy studies are necessary. Gene therapy involving the delivery of condensed tuberin via an AAV9 vector has shown promise in a mouse model of TSC [101].

## 6. Epigenopathies/Chromatinopathies

A growing number of ‘regulatory genes’ have been associated with LGS, including *CHD2*, *ARX*, and *PURA* [17]. These genes play key roles in regulating the expression of other genes and can be broadly categorized into transcription factors and epigenetic modifiers. Transcription factors bind specifically to DNA sequences, either promoting or blocking the recruitment of RNA polymerase [102]. Epigenetic modifiers, on the other hand, alter the histone protein matrix around which DNA is wrapped [103]. Epigenetic processes such as methylation, acetylation, ubiquitylation, and chromatin remodeling help regulate gene expression [104].

### CHD2

Chromodomain helicase DNA-binding protein 2 (CHD2) is crucial in modulating chromatin structure and is involved in processes like cell cycle regulation, development, and differentiation [105]. In CHD2-related disorders, seizures can include generalized tonic–clonic, myoclonic, atonic, atypical absence, focal, and myoclonic–atonic seizures, as well as epileptic spasms [106]. Other seizure types observed in these patients include myoclonic-atonic seizures, absence seizures with myoclonia, and infantile spasms.

Levetiracetam and valproate have been favorable treatments for patients with CHD2 mutations, but recent studies exploring add-on treatments with acetazolamide (ACZ) have shown remarkable efficacy [106]. In a clinical study, ACZ controlled photosensitive seizures in all patients—six of whom became seizure-free, while the remaining six had a reduction in seizure frequency of over 75% [107]. In a zebrafish model, ACZ exposure reduced ictal-like events by 72%, as shown by electrophysiological recordings of CHD2 knockdown larvae [107]. In comparison, fenfluramine also reduced ictal events, but the effect was less pronounced and not statistically significant, suggesting that ACZ’s effects may be specific to CHD2-related epilepsy [107]. However, the exact mechanisms behind ACZ’s efficacy in CHD2-related epilepsy remain unclear. Proposed mechanisms include inhibition of carbonic anhydrase, as well as effects on acid-sensing ion channels, R-type calcium channels, the water channel aquaporin-4, and large conductance calcium- and voltage-activated potassium channels (BK channels).

Research is ongoing to develop more targeted, mechanism-based therapies. One key approach is to use induced pluripotent stem cells (iPSCs) to create cortical organoids that model the cell-type-specific cellular and molecular mechanisms underlying *CHD2* mutations [108]. While several CHD2 mouse and zebrafish models have been developed, capturing spontaneous seizures has been challenging. Traditional AAV vectors for gene therapy are difficult to apply here due to the large size of the *CHD2* coding sequence (over 5 kb) [108].

One promising approach is the use of ASOs, which are small, single-stranded nucleotides that target specific RNA sequences to modify protein expression. For CHD2, the goal would be to increase CHD2 expression to overcome haploinsufficiency. Targeting the long non-coding RNA CHASERR with ASOs is being explored, as reducing the expression of CHASERR in mice has been shown to increase CHD2 mRNA and protein levels [109]. Another therapeutic strategy involves utilizing microRNA. In both of these emerging approaches, DNA methylation patterns could serve as a strong biomarker, given the distinctive methylation signature associated with *CHD2* haploinsufficiency [110].

## 7. Dysfunction in Neuronal Formation and Maturation

### CDKL5

*CDKL5* deficiency disorder (CDD) is a DEE characterized by early-onset, intractable epilepsy that may progress to LGS [111]. The CDKL5 protein is a kinase crucial for phosphorylation events that regulate signaling pathways, neurotransmitter release, synaptic plasticity, and neuronal development [112].

Despite being a rare disease, several precision therapeutics have been explored for CDD. Early attempts at treatment included ataluren, a compound promoting read-through of premature stop codons, which failed to improve seizure frequency or cognitive, motor, and behavioral outcomes in a small pilot study [113]. In contrast, ganaxolone, an allosteric modulator of synaptic and extrasynaptic GABAA receptors, became the first FDA-approved therapy for CDD-associated seizures [114]. A Phase 3 trial demonstrated that ganaxolone (n = 50) significantly reduced 28-day major motor seizure frequency compared to placebo (–30.7% vs. –6.9%; *p* = 0.0036) [114]. Manic/Hyperactive and Compulsive Behavior scores also improved, though the quality-of-life change (+2.6 points) was not statistically significant [115]. An ongoing clinical trial (NCT05249556) is evaluating ganaxolone in younger patients (6 months–2 years old). Fenfluramine has also shown promise in CDD. A small pilot study of six patients (five female; 83%) treated with 0.4–0.7 mg/kg/day (max: 26 mg/day) for a median of 5.3 months (range: 2–9 months) demonstrated a median 90% reduction in tonic–clonic seizure frequency (range: 86–100%) [116]. Tonic seizure frequency decreased by 50–60% in two patients [116]. One patient experienced fewer myoclonic seizures, while another developed new myoclonic seizures controlled with valproate [116]. A Phase 3 clinical trial is ongoing to examine the efficacy and safety of fenfluramine in CDD (NCT05064878). Soticlestat, a selective inhibitor of cholesterol 24-hydroxylase (CH24H), reduces neuronal hyperexcitability by modulating NMDA receptor activity. In the Phase II open-label ARCADE study, 12 patients received weight-adjusted soticlestat (≤300 mg/day twice daily), resulting in a 23.6% reduction in motor seizure frequency and a 30.5% reduction in total seizures [117]. Over 90% of patients exhibited functional improvements per their Clinical Global Impression-Clinician scores.

Recent studies suggest that CDKL5 phosphorylates the voltage-gated calcium channel Cav2.3 (*CACNA1E*) [118]. Loss of CDKL5-mediated phosphorylation results in Cav2.3 GOF properties, such as slower inactivation and enhanced cholinergic stimulation, leading to neuronal hyperexcitability. This mechanistic overlap with *CACNA1E* gain-of-function mutations in DEE69 suggests that CDD may be amenable to Cav2.3 inhibitors [118].

Experimental gene therapies for CDD include UX055 and CRISPR-based approaches. UX055 is an investigational AAV9-based gene therapy designed to deliver a functional *CDKL5* gene to neurons [119]. Preclinical studies showed motor and cognitive improvements in CDD mouse models and correction of neuronal hyperexcitability in patient-derived brain organoids. UX055 is administered via cerebrospinal fluid injection to target the central nervous system. Epigenetic editing strategies using dual AAV vectors aim to reactivate the silent, healthy *CDKL5* allele in females with CDD [120]. As CDD is X-linked, most female patients retain one functional but inactive *CDKL5* allele due to X-chromosome inactivation. Early preclinical studies demonstrated functional recovery in patient-derived brain organoids and mouse models, highlighting a promising avenue for future clinical trials.

## 8. Gene and Cell Therapy Strategies for Lennox–Gastaut Syndrome

Some gene-based therapies are already discussed in the individual sections, including ASO treatment (which uses antisense oligonucleotides to bind specific mRNA sequences and modify gene expression), siRNA (small interfering RNA, which induces degradation of target mRNA by incorporating into the RNA-induced silencing complex (RISC), causing sequence-specific gene silencing), miRNA (microRNA, which modulates gene expression by binding to the 3′ untranslated region (UTR) of mRNA, leading to translational repression or mRNA destabilization), gene replacement using AAV (Adeno-associated virus, which delivers functional copies of genes to correct deficiencies in specific tissues), and gene editing with CRISPR (which allows for precise modification of DNA sequences to correct genetic mutations or alter gene expression). However, additional approaches are being explored in the broader epilepsy field that could potentially be translated to LGS treatment.

A promising gene therapy strategy for LGS involves modulating neuronal excitability through ion channels. For example, overexpression of potassium channels like Kv1.1 using AAV vectors or CRISPR activation has shown significant antiepileptic effects in animal models of refractory focal neocortical and temporal lobe epilepsy [121]. This approach reduces neuronal excitability and raises the threshold required for neuronal firing, which could benefit LGS patients. Another gene therapy approach targets neuromodulatory peptides, particularly neuropeptide Y (NPY), which has shown promising results in reducing seizure frequency by up to 40% in various epilepsy models [122,123]. NPY binds to presynaptic Y2 or Y5 receptors, reducing glutamate release and addressing the excitatory-inhibitory imbalance in epilepsy [124]. Enhanced efficacy has been observed with combined therapies, such as using AAV vectors to deliver NPY and its receptors [124]. For LGS patients, targeted AAV-NPY injections into the thalamus could be particularly beneficial to target generalized seizures [125]. Additionally, epigenetic modulation strategies that modify DNA methylation and histone acetylation offer potential in restoring normal gene expression patterns and reducing seizure activity [126].

Cell-based approaches, such as the transplantation of mesenchymal stem cells (MSCs), have shown promise for treating drug-resistant epilepsy. Phase I/II clinical trials have demonstrated the safety and efficacy of MSCs administered alongside ASMs [127,128]. MSCs provide therapeutic effects through neuroprotection, immunomodulation, and neurogenesis support. They also have the ability to cross the blood–brain barrier, making intravenous delivery a viable treatment option. Combination approaches involving autologous bone marrow nucleated cells and MSCs could also be explored for LGS [129].

Encapsulated cell biodelivery (ECB) combines elements of gene and cell therapy by implanting capsules containing genetically modified cells that secrete therapeutic substances [130]. This technique allows for the local and sustained delivery of neurotrophic factors (e.g., GDNF, BDNF, galanin) while minimizing immune responses [131,132,133]. ECB offers the benefit of reversibility, as capsules can be removed if necessary, which is an important safety feature, particularly in younger patients with LGS.

Advanced techniques like optogenetics and chemogenetics provide unprecedented control over neural circuits. These approaches enable the temporal and spatial modulation of specific neural populations, potentially reducing seizure frequency or severity by selectively targeting hyperactive circuits while preserving normal brain function [134,135]. Another innovative technique is Designer Receptors Exclusively Activated by Designer Drugs (DREADDs), which allows for on-demand control of neural activity through pharmacologically inert compounds like clozapine-N-oxide (CNO) [136]. This approach has demonstrated efficacy in epilepsy models, with AAV-mediated expression of synthetic receptors in the dorsal hippocampus resulting in significantly reduced seizures in pilocarpine-induced epilepsy rats [137]. For LGS patients, this temporally controlled intervention could offer personalized treatment options, particularly for those experiencing seizure clusters (Table 2).

## 9. Future Directions in Precision Therapeutics for Lennox–Gastaut Syndrome

Precision therapy for LGS is still in its early stages, but advances in genomics and targeted therapeutics hold the potential to transform treatment (Table 3). However, significant challenges remain, including the genetic and mechanistic heterogeneity of LGS and the limited scalability of emerging therapies. Diagnostic inaccuracies further complicate care, as LGS is often overdiagnosed in individuals with other DEEs and underdiagnosed in patients whose seizure and EEG patterns evolve over time [138]. Overcoming these barriers will require integrated strategies that combine biological insights, innovative clinical trial designs, and equitable access to precision therapies [139,140,141,142,143,144,145,146]. A comprehensive approach to precision therapy in LGS must consider patient-specific clinical features, including cognitive and behavioral profiles. Standardized cognitive and behavioral assessments are essential for tailoring interventions and optimizing precision therapeutics. Additionally, EEG phenotyping and serial monitoring can refine treatment strategies by quantifying slow spike-wave and GPFA burden through longitudinal follow-up.

## 10. Conclusions

Precision therapeutics for LGS are in the early stages but evolving rapidly, driven by advancements in genetic, molecular, and network-level understanding. Despite significant challenges, the integration of biological insights with innovative clinical approaches holds promise for shifting LGS treatment from symptomatic management to targeted disease modification.

## Figures and Tables

**Table 1:** Molecular mechanisms and precision therapeutics in Lennox–Gastaut syndrome.

Molecular Mechanism	Gene/Protein	Precision Treatments
Channelopathies		
Sodium Channel	*SCN2A* (NaV1.2)	- Sodium channel blockers (carbamazepine, phenytoin) - Phrixotoxin-3 - Cenobamate - Relutrigine (PRAX-562) - Ketogenic diet - ASOs (elsunersen/PRAX-222)
	*SCN8A* (NaV1.6)	- Sodium channel blockers - Cenobamate - Relutrigine (PRAX-562) - NBI-921352 - Amitriptyline, carvedilol, nilvadipine - ASOs
Potassium Channel	*KCNQ2* (Kv7.2)	- Sodium channel blockers (carbamazepine, phenytoin) - Ezogabine (retigabine) - XEN496, XEN1101, BHV-7000 (in development)
	*KCNA2* (Kv1.2)	- 4-Aminopyridine
	*KCNT1*	- Quinidine - ASOs
Calcium Channel	*CACNA1A* (Cav2.1)	- Lamotrigine, topiramate, levetiracetam - Acetazolamide - Calcium channel blockers (flunarizine, verapamil) - Carbamazepine
Receptor- and Ligand-Mediated Dysfunction		
GABA Receptor	*GABRA1, GABRA2, GABRB2, GABRB3*	- Vinpocetine (for LOF variants) - Avoid vigabatrin for GOF variants
GABA Transporter	*SLC6A1* (GAT-1)	- Valproic acid, lamotrigine - Benzodiazepines - Ethosuximide - Ketogenic diet
Glutamate Receptor	*GRIN2B, GRIN1, GRIN2A, GRIN2D*	- Memantine - Radiprodil
	*GRIN2B, GRIN2A*	- L-serine
Synaptopathies		
Synaptic Vesicle Release	*STXBP1* (Munc18-1)	- Chemical chaperones (4-phenylbutyrate, sorbitol, trehalose) - Cannabidiol (CBD) - Levetiracetam - Ketogenic diet - Serotonergic drugs (clemizole, trazodone, fenfluramine) - miRNA therapies
AMPA Receptor Trafficking	*IQSEC2*	- AMPA receptor modulators (perampanel, methylphenidate, aniracetam) - Thyrotropin-releasing hormone pathway modulators
Synaptic Vesicle Endocytosis	*DNM1* (Dynamin 1)	- Ketogenic diet - Valproic acid, clobazam, vigabatrin - BMS-204352 - RNA interference (RNAi) - Combined RNAi + gene replacement
Cell Signaling Dysfunction		
mTOR Pathway	*TSC1, TSC2*	- Vigabatrin (preventative treatment) - mTOR inhibitors (everolimus, sirolimus) - Cannabidiol - AAV-mediated gene therapy
Epigenopathies/Chromatinopathies		
Chromatin Remodeling	*CHD2*	- Levetiracetam, valproate - Acetazolamide - Fenfluramine - ASOs targeting CHASERR - miRNA therapies
Dysfunction in Neuronal Formation and Maturation		
Neurodevelopmental Signaling	*CDKL5*	- Ganaxolone (FDA-approved) - Fenfluramine - Soticlestat - Cav2.3 inhibitors - Gene therapy (UX055) - CRISPR-based epigenetic editing

Abbreviations: GOF: Gain-of-function, LOF: loss-of-function, ASO: antisense oligonucleotide, AAV: Adeno-associated virus, mTOR: mechanistic target of rapamycin, miRNA: MicroRNA.

**Table 2:** Gene and cell therapy strategies for Lennox–Gastaut syndrome.

Gene Therapy Approach	Mechanism of Action	Examples	Significance to LGS
Ex Vivo Gene Therapy	Cells are removed, genetically modified outside the body, and reintroduced to the patient.	- CAR-T cell therapy - Gene-modified hematopoietic stem cell therapy for SCD and ADA-SCID	Could be used for cell-based therapies to repair brain circuitry. Encapsulated cell biodelivery (ECB), a specialized form of ex vivo gene therapy, has been used to deliver glial cell line-derived neurotrophic factor (GDNF) to the epileptic focus, preventing spontaneous recurrent seizures in an animal model.
In Vivo Gene Therapy (Non-Viral)	Direct gene delivery to the body using lipid nanoparticles or polymer-based systems.	- Lipid nanoparticles (e.g., mRNA vaccines) - Polymeric nanoparticles for DNA or RNA delivery - Approved products available for l-amino acid decarboxylase deficiency (AADC deficiency) and spinal muscular atrophy (SMA)	Could be used to introduce functional copies of genes like *CDKL5*, *DNM1*, etc., that are mutated in LGS. Another approach to reducing neuronal excitability involves the overexpression of neuromodulatory peptides such as neuropeptide Y (NPY) and galanin.
Epigenetic Modulation	Modifies gene expression through altering epigenetic marks like methylation or histone modifications.	- CRISPR-dCas9 for epigenetic regulation - Epigenetic drugs (e.g., DNMT inhibitors like azacitidine and decitabine, HDAC inhibitors like vorinostat and romidepsin, and EZH2 inhibitors like tazemetostat for specific cancers)	Can be used to modulate genes involved in epileptogenesis in LGS, especially genes like *CHD2* and *CDKL5*.
Optogenetic and Chemogenetic Approaches	Use light or chemicals to control gene expression or cellular activity in specific cells.	- MCO-010 to restore vision in retinitis pigmentosa	Network firing rates in human hippocampal slices, recorded using high-density microelectrode arrays under various hyperactivity-inducing conditions, were reduced through Adeno-associated virus-mediated optogenetic interventions. Potential for controlling abnormal neural circuits in LGS models, possibly reducing seizure frequency or severity.
CRISPR/Cas9 Gene Editing	Precise editing of genes to correct mutations at the DNA level, including base and prime editing.	- Prime editing for correcting point mutations - Base editing for converting one base pair to another - Casgevy for sickle cell anemia	Could be used to correct point mutations in *SCN2A*, CHD2, or other genes causing LGS, offering a long-term solution.
Antisense Oligonucleotides (ASOs)	Short DNA or RNA molecules designed to bind to specific RNA sequences, modulating gene expression or correcting splicing defects.	- Nusinersen (Spinraza) for SMA	*SCN2A*, *SCN8A*, *KCNT1*, *CHD2*
MicroRNAs (miRNAs)	Small non-coding RNA molecules that regulate gene expression by binding to target mRNA, leading to its degradation or translation inhibition.	- None commercially available	Research has identified that specific microRNAs can regulate STXBP1 expression, potentially impacting the levels of the Munc18-1 protein.
Small Interfering RNAs (siRNAs)	Short RNA molecules that degrade target mRNA, preventing gene expression.	- Patisiran for polyneuropathy caused by hereditary transthyretin-mediated amyloidosis and givosiran for acute hepatic porphyria.	Potential to silence harmful mutations or regulate overactive genes involved in LGS pathophysiology.

Abbreviations: CAR-T (chimeric antigen receptor T cell), SCD (sickle cell disease), ADA-SCID (adenosine deaminase–severe combined immunodeficiency), ECB (encapsulated cell biodelivery), GDNF (glial cell line-derived neurotrophic factor), AADC (l-amino acid decarboxylase), SMA (spinal muscular atrophy), DNMT (DNA methyltransferase), HDAC (histone deacetylase), EZH2 (enhancer of zeste homolog 2), ASOs (antisense oligonucleotides), miRNA (microRNA), siRNA (small interfering RNA).

**Table 3:** Future directions for precision therapy development in Lennox–Gastaut syndrome.

Strategic Area	Current Challenges	Proposed Approaches	Potential Impact
Diagnostic Accuracy	High rates of misdiagnosis	Computable phenotypes from EHR and EEG data	Improved patient stratification for trials
Molecular Subgrouping	Diverse etiologies converging on the LGS phenotype	Multi-omics integration (genomics, transcriptomics, proteomics), study the functional consequences of specific genetic mutations(in vitro electrophysiological techniques (patch-clamp) or in vivo models; investigate synaptic activity by measuring excitatory and inhibitory postsynaptic currents (EPSCs and IPSCs) in neuronal cultures or brain slices; animal models; neuroimaging studies; clinical and biomarker studies	Identification of shared targetable pathways
Drug Development	Focus on seizures rather than comorbidities	High-throughput screening of small molecules, gene therapies, or biologics that modulate the identified shared pathways	Holistic treatments addressing cognitive deficits
Gene-Targeted Therapies	Delivery challenges, off-target effects	Optimized ASOs, RNAi, and CRISPR-based strategies	Disease-modifying potential for monogenic causes
Neuromodulation	Limited personalization	Closed-loop DBS, RNS, targeted stimulation	Network-based interventions for drug-resistant cases
Clinical Trial Design	Traditional designs are inadequate for rare variants	Adaptive designs, basket trials, n-of-1 studies	Accelerated evaluation of precision treatments
Equitable Implementation	High costs, limited access	Expanded genetic testing access, public healthcare integration	Prevention of treatment disparities
